# Prostate cancer mortality outcomes and patterns of primary treatment for Aboriginal men in New South Wales, Australia

**DOI:** 10.1111/bju.12899

**Published:** 2015-04-01

**Authors:** Jennifer C. Rodger, Rajah Supramaniam, Alison J. Gibberd, David P. Smith, Bruce K. Armstrong, Anthony Dillon, Dianne L. O'Connell

**Affiliations:** ^1^ Cancer Research Division Cancer Council NSW Sydney NSW; ^2^ School of Public Health The University of Sydney Sydney NSW; ^3^ Institute for Positive Psychology and Education Australian Catholic University Sydney NSW; ^4^ Faculty of Medicine School of Public Health and Community Medicine University of New South Wales Sydney NSW; ^5^ Faculty of Health and Medicine School of Medicine and Public Health University of Newcastle Sydney NSW; ^6^ Griffith Health Institute Griffith University Gold Coast QLD Australia

**Keywords:** Aboriginal men, patterns of care, prostate cancer, mortality, outcomes, indigenous

## Abstract

**Objective:**

To compare prostate cancer mortality for Aboriginal and non‐Aboriginal men and to describe prostate cancer treatments received by Aboriginal men.

**Patients and methods:**

We analysed cancer registry records for all men diagnosed with prostate cancer in New South Wales (NSW) in 2001–2007 linked to hospital inpatient episodes and deaths. More detailed information on androgen‐deprivation therapy and radiotherapy was obtained from medical records for 87 NSW Aboriginal men diagnosed in 2000–2011. The main outcomes were primary treatment for, and death from, prostate cancer. Analysis included Cox proportional hazards regression and logistic regression.

**Results:**

There were 259 Aboriginal men among 35 214 prostate cancer cases diagnosed in 2001–2007. Age and spread of disease at diagnosis were similar for Aboriginal and non‐Aboriginal men. Prostate cancer mortality 5 years after diagnosis was higher for Aboriginal men (17.5%, 95% confidence interval (CI) 12.4–23.3) than non‐Aboriginal men (11.4%, 95% CI 11.0–11.8). Aboriginal men were 49% more likely to die from prostate cancer (hazard ratio 1.49, 95% CI 1.07–1.99) after adjusting for differences in demographic factors, stage at diagnosis, health access and comorbidities. Aboriginal men were less likely to have a prostatectomy for localised or regional cancer than non‐Aboriginal men (adjusted odds ratio 0.60, 95% CI 0.40–0.91). Of 87 Aboriginal men with full staging and treatment information, 60% were diagnosed with localised disease. Of these, 38% had a prostatectomy (± radiotherapy), 29% had radiotherapy only and 33% had neither.

**Conclusion:**

More research is required to explain differences in treatment and mortality for Aboriginal men with prostate cancer compared with non‐Aboriginal men. In the meantime, ongoing monitoring and efforts are needed to ensure Aboriginal men have equitable access to best care.

## Introduction

Prostate cancer is the most commonly diagnosed cancer and the third most common cause of cancer death in Australian men 1; it is also the most commonly diagnosed cancer in New South Wales (NSW) Aboriginal men 2. Compared with non‐Aboriginal men, reported prostate cancer incidence rates for Australian Aboriginal men appear to be lower 2, 3, 4, 5, 6, 7, possibly due to lower uptake of PSA testing 8, 9. Limited research suggests that survival after diagnosis is poorer for Aboriginal men 2, 10, 11, which may be due to differences in the timing of diagnosis and access to prostate cancer treatment between Aboriginal and non‐Aboriginal men.

The optimal management of localised prostate cancer is difficult to define as evidence showing a clear survival benefit of one treatment over another is sparse 12. It is unknown whether treatment factors explain higher mortality for Aboriginal men with prostate cancer, as little is known about their treatment patterns. One older Western Australian study identified lower rates of surgical treatment for Aboriginal men compared with non‐Aboriginal men with prostate cancer 13.

The aim of the present study was to investigate differences in prostate cancer‐specific mortality in Aboriginal and non‐Aboriginal men diagnosed with prostate cancer, and to explore how various factors, including treatment patterns, might contribute to mortality differences. An additional objective was to describe in more detail the staging and primary treatment for Aboriginal men diagnosed with prostate cancer through medical record review.

We respectfully use the descriptor ‘Aboriginal’ throughout this paper to refer to the original people of Australia and their descendants, as endorsed by the Aboriginal Health and Medical Research Council of NSW and NSW Health 2004 14.

## Patients and Methods

Linked datasets from two studies were analysed: the NSW Population‐wide Study (2001–2007), which included NSW Central Cancer Registry (CCR) records of incident prostate cancers in NSW linked to hospital and death records, and the Patterns of Primary Treatment Study (2000–2011), which was an audit of the medical records of Aboriginal men diagnosed with prostate cancer linked to CCR and hospital records. Eligible patients for both studies were diagnosed with primary prostate cancer (ICD‐0‐3 topography code ‘C61’ and morphology codes with a suffix of ‘3’), aged ≥18 years and resident in NSW at diagnosis.

### NSW Population‐Wide Study

#### Data sources

Demographic and disease information for all eligible men diagnosed with prostate cancer in 2001–2007 (37 271 men) was obtained from the CCR. Cases were matched by the Centre for Health Record Linkage (CHeReL) with inpatient records from the NSW Admitted Patient Data Collection (APDC), death records from the NSW Registry of Births, Deaths and Marriages (RBDM), and cause of death data from the Australian Bureau of Statistics (ABS). As this was a study of care and outcomes for men with prostate cancer, we excluded 375 men with death certificate or autopsy notification only and an additional 1682 men who did not link to APDC records. In this analysis a man with prostate cancer was determined to be Aboriginal if he was listed as Aboriginal in any of his records.

#### Variables for analysis

The CCR data included month and year of diagnosis, age and spread of disease at diagnosis categorised as localised, regional, distant and unknown. Based on the Local Government Area of residence at diagnosis men were assigned to geographical location categories according to the Accessibility/Remoteness Index for Australia (ARIA+) 15 and to tertiles of socioeconomic disadvantage according to the ABS Socio‐Economic Indexes for Areas (SEIFA) Index of Relative Socio‐Economic Advantage and Disadvantage 16, 17.

Analysis of treatment was restricted to surgical treatment as the APDC has been shown to be an incomplete source of information for other treatment methods 18. Non‐cancer conditions described in the Charlson Comorbidity Index 19 were obtained from the APDC diagnosis codes for any hospital admission between 12 months before and 6 months after prostate cancer diagnosis. The APDC does not contain detailed clinical information, such as tumour characteristics, PSA levels, whether lymph node dissection was performed, and whether radiotherapy was received.

#### Statistical methods

All analyses were performed using SAS statistical software (release 9.3; SAS Institute Inc., Cary North Carolina) and R 2.15.1 20.

Prostate cancer‐specific mortality was analysed using cumulative incidence curves 21 and Cox proportional hazards regression models 22. The unadjusted model included Aboriginal status as the sole independent variable. The fully adjusted model included the following additional variables: age at diagnosis, year of diagnosis, spread of disease at diagnosis, comorbidities, socioeconomic disadvantage, place of residence and whether treated with prostatectomy. Follow‐up was censored at 31 December 2008 for all surviving men and all non‐prostate cancer deaths were censored at the date of death. We tested for interactions between Aboriginal status and other variables.

Logistic regression was used to identify variables that significantly influenced the odds of having a prostatectomy within 12 months of diagnosis. The fully adjusted model included the following variables: Aboriginal status, age at diagnosis, year of diagnosis, spread of disease, comorbidities, socioeconomic disadvantage and place of residence. We tested for interactions between Aboriginal status and the other variables.

### Patterns of Primary Treatment Study

#### Study design

We collected medical records for Aboriginal people resident in NSW diagnosed with any cancer in 2000–2011. Data were collected from 23 public hospitals and three clinical cancer registries for 1324 people of whom 87 were Aboriginal men with prostate cancer. We estimate there were about 407 Aboriginal men diagnosed with prostate cancer in NSW in that period. Records for these 87 men were linked by the CHeReL to CCR and APDC records for additional information.

#### Variables for analysis

Information collected included age, year of diagnosis, spread of disease, place of residence and comorbidities. Tertiles of socioeconomic disadvantage and ARIA+ category were assigned using postcode of residence. PSA levels and Gleason score at diagnosis were recorded. We also recorded start and end dates for treatments received [prostatectomy, radiotherapy and androgen‐deprivation therapy (ADT)]. We collected and merged data for men who attended multiple hospitals.

## Results

### NSW Population‐Wide Study

#### Participant characteristics

Of the 35 214 eligible men, 259 (0.7%) were identified as Aboriginal (Table 1). Aboriginal and non‐Aboriginal men had a similar age and spread of disease at diagnosis. Aboriginal men were more likely to live outside major cities and in socioeconomically disadvantaged areas. They were also more likely than non‐Aboriginal men to have diabetes, cardiovascular disease, chronic pulmonary disease or renal disease at the time of prostate cancer diagnosis.

**Table 1:** Comparison of Aboriginal and non‐Aboriginal men diagnosed with prostate cancer in NSW, 2001–2007 (*N* = 35 214)

Variable	Aboriginal, *N* (%)	Non‐Aboriginal, *N* (%)	*P*
All men diagnosed	259	34955	
Age at diagnosis (years):			0.115
18–59	42 (16)	5949 (17)	
60–69	100 (39)	12097 (35)	
70–79	90 (35)	11473 (33)	
≥80	27 (10)	5436 (16)	
Year of diagnosis:			0.499
2001	22 (8)	3697 (11)	
2002	32 (12)	3985 (11)	
2003	28 (11)	4424 (13)	
2004	37 (14)	5204 (15)	
2005	50 (19)	5643 (16)	
2006	49 (19)	5801 (17)	
2007	41 (16)	6201 (18)	
Place of residence at diagnosis*			<0.001
Major cities	112 (43)	23521 (67)	
Inner regional	85 (33)	8693 (25)	
Rural^†^	62 (24)	2741 (8)	
Socioeconomic disadvantage tertile*:			<0.001
Least disadvantaged	36 (14)	12088 (35)	
Moderately disadvantaged	80 (31)	10069 (29)	
Most disadvantaged	143 (55)	12798 (37)	
Comorbidities:			
Diabetes	47 (18)	3524 (10)	<0.001
Cardiovascular disease^‡^	39 (15)	2970 (9)	<0.001
Chronic pulmonary disease	31 (12)	1755 (5)	<0.001
Renal disease	15 (6)	995 (3)	0.005
Other comorbid conditions	19 (7)	1685 (5)	0.062
No known comorbidities	130 (58)	22216 (74)	<0.001
No comorbidity information available	36 (14)	5006 (14)	0.847
Extent of disease:			0.289
Localised	120 (46)	17708 (51)	
Regional	19 (7)	2034 (6)	
Distant	15 (6)	1445 (4)	
Unknown	105 (41)	13768 (39)	
Prostatectomy 12 months post‐diagnosis by stage			
Localised	36 (30)	7684 (43)	0.003
Regional	9 (47)	1251 (62)	0.208

*Based on Local Government Area of place of residence; ^†^‘Rural’ includes outer regional, remote and very remote; ^‡^Myocardial infarction, congestive heart failure, peripheral vascular disease or cerebrovascular disease.

#### Mortality

**Figure 1:**
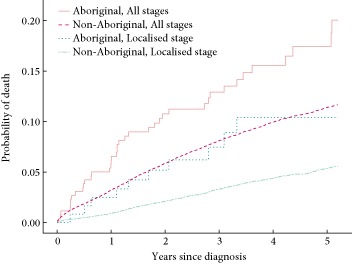
Cumulative probability of death from prostate cancer for all stages and localised stage for Aboriginal and non‐Aboriginal men with prostate cancer in NSW, 2001–2007 (*N* = 35 214).

The crude probability of death from prostate cancer by 5 years after diagnosis was 53% higher for Aboriginal men (17.5%, 95% CI 12.4–23.3) compared with non‐Aboriginal men (11.4%, 95% CI 11.0–11.8) (Fig. 1). A similar difference in mortality was seen when the analysis was limited to patients with localised disease. The unadjusted hazard ratio (HR) was 1.79 (95% CI 1.29–2.40) and the fully adjusted HR was 1.49 (95% CI 1.07–1.99).

There was a significant interaction between spread of disease and Aboriginal status (*P* for interaction = 0.044). After adjusting for all covariates the HR for prostate cancer death in Aboriginal men, relative to non‐Aboriginal men, was 1.83 (95% CI 0.92–3.24) for localised disease, 3.66 (95% CI 1.13–8.63) for regional disease, 0.75 (95% CI 0.34–1.41) for distant disease and 1.81(95% CI 1.11–2.76) for men with unknown spread of disease.

#### Surgical treatment

Aboriginal men were less likely to have a prostatectomy than non‐Aboriginal men for both localised (30% vs 43%) and regional (47% vs 62%) disease, although the difference for regional disease was not statistically significant (Table 1). The median number of days from diagnosis to prostatectomy was not significantly different between Aboriginal (67 days) and non‐Aboriginal men (65 days).

Aboriginal men were significantly less likely than non‐Aboriginal men to receive prostatectomy for localised or regional disease after accounting for differences in age at diagnosis, year of diagnosis, spread of disease, comorbidities, socioeconomic disadvantage and place of residence at diagnosis [odds ratio (OR) 0.60, 95% CI 0.40–0.91]. No interaction between Aboriginal status and any other variable had a *P* < 0.05.

### Patterns of Primary Treatment Study

#### Participant characteristics

Most of the 87 Aboriginal men in this study (56%) were aged 60–69 years when diagnosed (Table 2). In all, 60% of the men were diagnosed with localised prostate cancer, 57% had a Gleason score of ≥7, 40% had PSA levels of at ≥10 ng/mL, and 23% had PSA levels of ≥20 ng/mL. There were 60% of men living in major cities or inner regional areas. Two‐thirds of the Aboriginal men (66%) lived in areas that were in the most socioeconomically disadvantaged tertile.

**Table 2:** Sociodemographic and disease characteristics of the 87 Aboriginal men included in The Patterns of Primary Treatment Study

Characteristic	*N* (%)
Age at diagnosis (years)	
18–59	18 (21)
60–69	49 (56)
70–79	16 (18)
≥80	4 (5)
Year of diagnosis	
2000–2003	15 (17)
2004–2007	48 (55)
2008–2011	24 (28)
Place of residence at diagnosis*	
Major cities	35 (40)
Inner regional	17 (20)
Rural^†^	35 (40)
Socioeconomic disadvantage tertile*	
Least disadvantaged	8 (9)
Moderately disadvantaged	22 (25)
Most disadvantaged	57 (66)
Comorbidities	
No comorbidity	27 (31)
Diabetes	20 (23)
Cardiovascular disease	21 (24)
Chronic pulmonary disease	12 (14)
Renal disease	5 (6)
Unknown	2 (2)
Stage at diagnosis:	
T1–T2	52 (60)
T3	11 (13)
T4	8 (9)
Unknown	16 (18)
Gleason score at diagnosis	
2–6	23 (26)
7	30 (34)
8–10	20 (23)
Unknown	14 (16)
PSA at diagnosis (ng/mL)	
0–3.9	3 (3)
4–9.9	30 (34)
10–19.9	15 (17)
≥20	20 (23)
Unknown	19 (22)

*Based on postcode; ^†^‘Rural’ includes outer regional, remote and very remote areas.

The men in the ‘Patterns of Primary Treatment Study’ were on average younger, more likely to live in rural and more disadvantaged areas and had later stage disease than Aboriginal men in the NSW Population‐wide Study (Tables 1 and 2).

#### Primary treatment

It is notable that 41% of the Aboriginal men received neither surgery nor radiotherapy (Table 3) for their prostate cancer within 12 months of diagnosis. While this proportion was greater in men with non‐localised (47%) or unknown stage cancer (63%), it was still quite high in men with localised cancer (33%) and did not vary appreciably by the pathological risk rating of the localised cancer. Of the men with localised disease who were treated within 12 months, 38% had a prostatectomy, 28% had radiotherapy (± ADT) and 19% had ADT only. The proportion treated with radiotherapy (± ADT) increased with increasing pathological risk rating from 9% in men with low‐risk cancers to 53% with high‐risk cancers. ADT was the only treatment received by 37% of men with non‐localised disease and for a quarter (25%) of men with unknown spread of disease.

**Table 3:** Primary treatment received by Aboriginal men with prostate cancer within 12 months of diagnosis in NSW, 2000–2011 (N = 87)

Extent of disease (*N*)	Surgery (± radiotherapy ± ADT), *N*	Radiotherapy (± ADT), *N*	No surgery or radiotherapy, *N*		
			ADT only	No ADT	ADT unknown
Localised (52)*	20	15	10	5	2
Low risk (11)	6	1	–	–	–
Intermediate risk (22)	10	6	–	–	–
High risk (15)	2	8	–	–	–
Not localised (19)	2	8	7	2	0
Unknown (16)	5	1	4	3	3

*The risk could not be determined for four of the 52 men with localised spread due to missing PSA levels and/or Gleason scores. Information on ADT not provided due to small numbers. Low risk: PSA level <10.0 ng/mL and Gleason score ≤6; intermediate risk: PSA level ≥10– < 20 ng/mL or Gleason = 7; high risk: PSA level ≥ 20.0 ng/mL or Gleason score ≥8.

Of the 36 men who received neither prostatectomy nor radiotherapy, most (58%) were treated with ADT within 12 months of diagnosis, while 28% men did not receive ADT and five had no information available.

## Discussion

The risk of death from prostate cancer was higher in Aboriginal men than non‐Aboriginal men diagnosed with prostate cancer in NSW. Differences in age at and year of diagnosis, spread of disease, treatment with prostatectomy, place of residence, socioeconomic disadvantage and comorbidities did not fully explain this disparity. We found that Aboriginal men were significantly less likely than non‐Aboriginal men to receive prostatectomy for localised or regional disease after accounting for differences in demographic, disease, and health‐access factors and comorbidities. We also found that 33% of Aboriginal men diagnosed with localised prostate cancer received neither surgery nor radiotherapy early in the disease course, and this was true regardless of the pathologically assessed disease recurrence risk. In comparison, in a 2000–2002 study of NSW men aged <70 years with localised prostate cancer, 20% received neither surgery nor radiotherapy 23; an observation that is consistent with, although not indicative of, a higher rate of potentially curative treatment in all NSW men than in Aboriginal NSW men (men in the 2000–2002 NSW study had a younger average age at diagnosis and mainly earlier treatment period). The lower rate of prostatectomy for prostate cancer in Aboriginal men compared with other men in the NSW Population‐wide Study supports this possibility. It is possible that a proportion of men for whom we have no treatment information were under active surveillance; however, we were not able to determine this conclusively from the medical records.

Diagnosis with more aggressive disease is another possible explanation for a higher risk of death in Aboriginal men. Aboriginal men in our Patterns of Primary Treatment Study had a higher median PSA level at diagnosis (10.9 ng/mL) than found in other Australian population studies (NSW 6.8 ng/mL 23 and Victoria 7.7 ng/mL 24). However, as the Aboriginal men had a similar median Gleason score (7.0) to those in other Australian studies (NSW 6.5 23 and Victoria 6.9 24), more aggressive disease is unlikely to fully explain the differences seen. Differences in the adequacy of follow‐up, ongoing monitoring, and adjuvant ADT or radiotherapy may have contributed to the mortality difference, but our present data cannot address them.

It is possible that the mortality difference between Aboriginal and non‐Aboriginal men is more apparent than real. Previous research suggests that routine PSA testing in asymptomatic men was less common in Aboriginal men than non‐Aboriginal men 8, 9. Higher PSA testing rates in non‐Aboriginal men might, by earlier diagnosis, create lead‐time bias sufficient to give a false impression that non‐Aboriginal men survive longer. Similarly, as PSA testing identifies indolent cancers that may otherwise remain undiagnosed for long periods of time, if not indefinitely 25, length bias might also give the appearance of better outcomes in non‐Aboriginal men. We also cannot exclude that some of the observed difference in mortality is due to residual confounding, particularly by comorbidities, which are more common in Aboriginal men and may limit their treatment options.

While the present NSW Population‐wide Study is the largest and most comprehensive study of mortality and treatment patterns of Australian Aboriginal men with prostate cancer, it does have limitations. Firstly, the identification of Aboriginal men was based on classifications in the source datasets and may not have correctly identified all individuals. As 98% of the Australian population is non‐Aboriginal, the chance of positive misclassification is low. We attempted to minimise Aboriginal under identification by using any recording of Aboriginal status in any linked records. The high proportion of men classified as ‘unknown stage’ by the CCR (41% of Aboriginal and 39% of non‐Aboriginal men) is another limitation to our present findings. It cannot be assumed that the disease characteristics of men in the ‘unknown stage’ group are similar in Aboriginal and non‐Aboriginal men or that their expected outcomes would be similar. Using administrative datasets means that detailed clinical information that could be used to stratify the men into risk categories or describe their PSA testing history was not available. A further limitation is the potential under reporting of comorbidities in the hospital records, although diabetes has been shown to be reasonably reliably recorded 18, it is probable that the comorbidities are under reported in the APDC and were unknown for men who were not admitted to hospital in the period 12 months before, and up to 6 months after their prostate cancer diagnosis. Finally the ABS acknowledges that the number of Aboriginal deaths is underestimated due to difficulties in identifying Aboriginal people after death with 1.3% of deaths (≈1800 deaths in 2008) not having Indigenous status recorded 26.

The Patterns of Primary Treatment Study has some limitations. While the present study, to our knowledge, is the most detailed study of Aboriginal patterns of prostate cancer care conducted, it was small and had limited coverage of all hospitals that treat prostate cancer, and thus may not be representative of the treatment of all NSW Aboriginal men with prostate cancer.

Previous research into prostate cancer treatment patterns for Aboriginal men is limited to one older Western Australian study, which also showed that Aboriginal men had lower rates of prostatectomy than non‐Aboriginal men 13. International studies of prostate cancer treatment and survival in Indigenous populations have also reported broadly similar findings. Data from New Zealand showed that prostate cancer mortality rates for Māori men diagnosed with prostate cancer were significantly higher than for non‐Māori men 27, with the differences in survival remaining even after adjustment for age and Gleason score 27. Another study found that Māori men with prostate cancer in Wellington, New Zealand, were more likely to receive external beam radiotherapy than non‐Māori men, possibly because this was the only publicly funded treatment option, and because surgery may not have been appropriate for many Māori men due to comorbidities 28. Similarly, Canadian First Nations men living on‐reserve were found to have higher prostate cancer mortality than the general Canadian population 29.

Larger and more detailed population‐based studies of Australian Aboriginal men with prostate cancer are needed to investigate if the mortality disparity we observed is due to differences in diagnosis, aggressiveness of disease, treatment received or other factors; and if there is a treatment disparity, why. Ideally, ongoing population‐based monitoring of prostate cancer treatment and outcomes in NSW, which is already available in the Australian state of Victoria 24, would allow more up to date and comprehensive surveillance information for improving health services. Increasing use of technologies, e.g. MRI, to improve the diagnosis, monitoring and treatment of prostate cancer 30 is also unlikely to benefit Aboriginal men in NSW unless specific efforts are made to ensure access regardless of place of residence or socioeconomic disadvantage. Psychosocial research might also be informative; more general studies have identified social, financial or cultural barriers affecting treatment choices and treatment effectiveness for NSW Aboriginal people such as a lack of social inclusion and health literacy 31, 32; however, more work is needed to understand the specific needs of Aboriginal men with prostate cancer.

In conclusion, Australian Aboriginal men diagnosed with prostate cancer are at higher risk of subsequent death from prostate cancer than other Australian men. Adjustment for demographic, disease stage at diagnosis, having surgical treatment or comorbidities did not explain this disparity. However, Aboriginal men appear less likely to have surgery or radiotherapy when diagnosed with prostate cancer than other Australian men. Further research on, and ongoing population‐based monitoring of, prostate cancer treatment and outcomes are required to understand and address the reasons for these disparities. In the meantime, efforts are needed to ensure Aboriginal men have equitable access to best care when diagnosed with prostate cancer.

## Funding and Ethics Approval

This work was produced as part of the APOCC, which was funded by a National Health and MRC Health Services grant (Application Ref: 440202). Linkage of the ‘Patterns of Primary Treatment’ data set to the APDC was funded by a Cancer Institute NSW Epidemiology Linkage Grant (10/EPI/2‐05).

The ‘Patterns of Primary Treatment Study’ was approved by the Human Research Ethics Committees of the Royal Prince Alfred Hospital and the AH&MRC. Local Regional Governance Offices granted site‐specific approval for data collection in participating hospitals and CCRs. The ‘NSW Population‐wide Study’ and linkage of the ‘Patterns of Primary Treatment Study’ to the population datasets were approved by the NSW Population and Health Services Research Ethics Committee and the Human Research Ethics Committee of the AH&MRC.

## Conflict of Interest

All authors have no conflicts of interest to declare.

**Abbreviations**
ABS: Australian Bureau of Statistics
ADT: androgen‐deprivation therapy
AH&MRC: Aboriginal Health and Medical Research Council of NSW
APDC: Admitted Patient Data Collection
APOCC: Aboriginal Patterns of Cancer Care Project
ARIA+: Accessibility/Remoteness Index for Australia
CCR: Central Cancer Registry
CHeReL: Centre for Health Record Linkage
HR: hazard ratio
NSW: New South Wales
OR: odds ratio
